# Perceptions of and interest in HIV pre-exposure prophylaxis use among adolescent girls and young women in Lilongwe, Malawi

**DOI:** 10.1371/journal.pone.0226062

**Published:** 2020-01-13

**Authors:** Bertha Maseko, Lauren M. Hill, Twambilile Phanga, Nivedita Bhushan, Dhrutika Vansia, Linda Kamtsendero, Audrey E. Pettifor, Linda-Gail Bekker, Mina C. Hosseinipour, Nora E. Rosenberg

**Affiliations:** 1 UNC Project-Malawi, Lilongwe, Malawi; 2 Department of Health Behavior, University of North Carolina, Chapel Hill, North Carolina, United States of America; 3 Cecil G. Sheps Center for Health Services Research, University of North Carolina, Chapel Hill, North Carolina, United States of America; 4 Institute for Global Health and Infectious Diseases, School of Medicine, University of North Carolina at Chapel Hill, Chapel Hill, North Carolina, United States of America; 5 Department of Epidemiology, University of North Carolina, Chapel Hill, North Carolina, United States of America; 6 Desmond Tutu HIV Centre, UCT, Faculty of Health Sciences, Cape Town, South Africa; Sefako Makgatho Health Sciences University, SOUTH AFRICA

## Abstract

**Background:**

Oral Pre-Exposure Prophylaxis (PrEP) is an effective HIV prevention strategy for adherent users. Adolescent girls and young women (AGYW) in sub-Saharan Africa may particularly benefit from PrEP because of the disproportionate burden of HIV in this group. Understanding potential users’ perceptions of and interest in using PrEP is critical to promote the utilization of PrEP by individuals at risk of HIV.

**Methods:**

This qualitative investigation of AGYW’s knowledge of and interest in PrEP use was conducted in the context of Girl Power, a quasi-experimental cohort study comparing four models of service delivery at four health centers in Lilongwe, Malawi. We conducted individual in-depth interviews (IDIs) with 40 HIV-negative AGYW ages 15–24 years old six months after enrolment in the parent study. An explanation of PrEP was provided to participants. Interview topics included participants’ prior knowledge of, interest in, concerns about, and delivery preferences for PrEP. Analysis consisted of structural coding of interview transcripts corresponding to interview topics, summary of responses within these topics, and identification and description of emerging themes within each topic.

**Results:**

None of the AGYW had knowledge of PrEP prior to the IDIs, but once explained, a majority expressed an interest in using it due to inconsistencies in condom use, condom use errors, their own or their partners’ concurrent sexual partnerships, and rape. Most AGYW hoped that PrEP would be available in youth-friendly sections of health centers for easy access and youth-friendly counselling. They suggested that discrete packaging of PrEP would be needed to ensure user privacy. Concerns about relationship destabilization and accusations of promiscuity were raised as potential barriers to use.

**Conclusion:**

General interest in PrEP among AGYW was high. Discrete packaging and access to youth-friendly PrEP delivery modalities may facilitate the utilization of PrEP as a prevention strategy among sexually active AGYW. Attention to potential negative reactions from partners and community members to PrEP use will be needed when introducing PrEP to this population.

## Introduction

In the past three decades, many populations in sub-Saharan Africa have been greatly impacted by the HIV epidemic, and adolescent girls and young women (AGYW) are no exception. Young women are at very high risk of acquiring HIV [1,2]; In Malawi, new HIV infections among AGYW ages 15–24 are more than twice those among young men of the same age [3,4]. Approximately 12,500 new HIV infections occur among young people ages 15–24 years annually; 70 percent of these new HIV infections are among young women [5]. AGYW in this context remain at elevated risk for infection due to biological, social, and behaviour factors[5]. While effective strategies exist to promote the use of condoms [6], condom use is primarily male-controlled and often affords women only a limited ability to protect their sexual health because of the need to negotiate condom use with their sexual partners.

Oral Pre-Exposure Prophylaxis (PrEP), an effective user-controlled method of HIV prevention, holds great promise for this population. Oral PrEP, the use of combination antiretroviral therapy for HIV prevention, is 90% effective in preventing HIV when taken with good adherence [7,8],. Because PrEP pill-taking is self-controlled and requires no specific action or negotiation at the time of sex, this method holds particular promise as an HIV prevention method for AGYW who may have limited powers to negotiate condom use.

World Health Organization (WHO) guidelines recommended PrEP use among at-risk populations worldwide including AGYW in high burden settings [9]. Following the WHO recommendation, many countries including Malawi are beginning to pilot implementation of PrEP to AGYW and other key populations. As Malawi introduces PrEP to this important population, we need to understand more about AGYW’s potential interest in and for PrEP use to inform the delivery of this new prevention method. To this end, we conducted a qualitative study to understand knowledge of, interest in, concerns about, and delivery preferences for PrEP among sexually active AGYW enrolled in a prospective study investigating models of AGYW service delivery in Lilongwe, Malawi. With this information, we hope to understand how to tailor PrEP education and delivery to inform the successful rollout of PrEP to this vulnerable population in Malawi and similar settings.

## Methods

### Study context

The data presented here were collected in the context of Girl Power, a quasi-experimental cohort study comparing four models of service delivery at four health centers in Lilongwe, conducted between February 2016 and August 2017. Sexually active AGYW ages 15–24 years were enrolled in the parent study [10,11]. Four models of service-delivery were compared in four separate clinics: Model 1) standard of care, Model 2) Integrated youth-friendly health services; Model 3) Model 2 plus a small-group behavioral intervention on various health and social topics; and Model 4) Model 3 plus a cash transfer ($5.50/month) that was conditional on attending each behavioral intervention session. The Girl Power intervention clinics (Models 2–4) offered a range of sexual reproductive health services (SRH) that were integrated, offered in youth-dedicated areas, and staffed by providers trained in youth-friendly health services. The services included: HIV testing, syndromic management of sexually transmitted infections (STI), family planning, and condom distribution. These services were offered to all AGYW regardless of their eligibility to participate in the study. The main trial results have been published previously [10, 11]. PrEP was not provided in this study, nor was it available in government clinics at the time of the study.

AGYW were eligible to participate if they were 15–24 years old, from the clinics’ catchment areas, and willing to provide locator information. Sexually active AGYW were purposively recruited from urban and semi urban clinic defined catchment areas through a combination of community outreach, participant referral, and self-referral. Community outreach consisted of peer educators visiting socioeconomically disadvantaged parts of the catchment area. They engaged AGYW in one-on-one conversations about what study participation entailed. AGYW who enrolled were then provided with 3 invitations to invite friends (participant referral). AGYW who learned of the study through other sources in the communities such as or friend, could also enroll (self-referral). Recruitment from within the clinic itself was discouraged in order to prevent selection biases. One thousand participants ages 15–24 years old who reported being sexually active and living within the study clinic catchment area were enrolled in the trial, with 250 participants from each health facility.

### Qualitative study participants and procedures

The qualitative sub-study presented here was undertaken to elicit participants’ opinions and perceptions of PrEP and its delivery if it were to be implemented in Malawi. We aimed to answer three primary research questions through these interviews: 1) *What are the reasons for which AGYW might be interested in using PrEP*?; 2) *What are the reasons for which AGYW might be concerned about using PrEP*?; 3) *What methods and features of PrEP delivery are important to the acceptability of PrEP among AGYW*? Because many at-risk AGYW may not be highly engaged in healthcare, we sought to understand whether PrEP interest and preferences for delivery would differ by high-engagement (“good care-seekers”), and low-engagement (“poor care seekers”) and also to make sure we were not selectively getting responses from good care-seekers. Both groups’ interests will have an impact on considering future PrEP implementation and management of programs tailored to AGYW health seeking behaviours. To understand these issues by level of healthcare engagement, we purposively sampled 40 qualitative study participants who were classified as good care-seekers (N = 20) or poor-care-seekers (N = 20). The level of care seeking was based on data from monthly or quarterly visits schedules for care services they received within the past 6 months in the Girl Power study. We purposely sampled 10 participants from each of the four study clinics: five good care seekers and five poor care seekers. We defined good care seekers as participants who obtained multiple services in the past 6 months, while poor care seekers were those who enrolled and received one or no services in the past 6 months. All 40 participants recruited for interview were sexually active.

### Data collection

Individual in-depth interviews (IDIs) with the 40 participants occurred between November 2016 and February 2017. All participants completed informed consent in the main study prior to the interview. Malawian qualitative research specialists trained in qualitative research methods and fluent in both English and Chichewa, the local language, carried out qualitative interviews. The IDIs were conducted in Chichewa using a semi-structured interview guide, and took place in private clinic locations where conversations could not be overheard. PrEP was explained briefly during the in-depth interviews. To start, the interviewer introduced PrEP by asking participants if they had previously heard of PrEP and then provided an explanation of PrEP as follows:

*“PrEP is a way for people who do not have HIV to prevent it*. *PrEP are pills you take every day that contain some of the same medicine used to treat people who already have HIV*. *If you have PrEP medicine in your body and you are exposed to the HIV virus*, *the PrEP medicine in your body will stop the virus from being able to multiply in your body*. *People who are HIV-positive cannot take PrEP instead of ARVs and people who are HIV-negative cannot take ARVs instead of PrEP*.”

Interview topics corresponding to the research questions stated above pertained to women’s interest in, concerns about, and delivery preferences for PrEP. Specific interview questions for each of these topics are listed in Table 1. Neutral probes were used to gather detailed responses and elicit illustrative examples where necessary. This gave the participant the chance to elaborate on information that she felt important to tell the interviewer. Conversations were audio-recorded with permission to ensure all data were captured verbatim. Each interview contained additional general questions about sexual behaviour and care seeking, not reflected in this analysis, and typically lasted one hour (60 minutes). A small incentive ($3) was provided at the six-month study visit, when the IDIs were conducted. Following each interview, the research specialist wrote a summary of the interview and shared this with the PI. This allowed for routine debriefing in order to identify emerging themes. Later, a team of research assistants transcribed each interview directly into English translating from Chichewa audio recordings.

**Table 1:** Research and interview questions.

Research Question	Interview Questions
**1) What are the reasons for which AGYW might be interested in using PrEP?**	- How interested would you be in PrEP if it were available? Why? - What about it would be appealing to you? - How would it compare to using male condoms?
**2) What are the reasons for which AGYW might be concerned about using PrEP?**	- Are there any reasons you would be concerned about using PrEP? - How might using PrEP affect your romantic relationships? - Would you still be interested if you experienced side effects, like dizziness?
**3) What methods and features of PrEP delivery are important to the acceptability of PrEP among AGYW?**	- How would you prefer that PrEP be packaged? - Where would you prefer to receive PrEP? At a Girl Power clinic? Elsewhere? - What type of support would you want to help you take PrEP daily?

### Data analysis

We began analysis of the interviews by reading the summaries and full transcripts until we became familiar with their content. We developed a codebook beginning with structural codes corresponding to each interview topic and adding codes representing emerging themes identified through an iterative process: Participant responses were summarized within each topic and themes were noted within topics; codes corresponding to each emergent theme were applied to the data and the content relevant to each theme was summarized and sub-themes were noted [12]. Thematic codes were discussed with the full research team until consensus was reached for all codes. Each transcript was double-coded to ensure quality and consistency using NVivo version 11 software. We used Cohen’s Kappa to measure interrater reliability. All transcripts were double coded by two independent coders the researcher and coding compared. For transcripts with less than 80% agreement we reviewed the discrepancies together and re-coded. Responses relating to each interview topic and emerging theme were summarized across participants and differences between individuals and among sub-groups of participants were noted.

### Ethical considerations

Prior to the implementation of the study, the study team obtained approval from University of North Carolina Chapel Hill Institutional Review Board and the Malawi National Health Sciences Research Committee. Written, informed consent to participate in an IDI was obtained from all participants age 18 and above on entry to the parent study. For participants considered minors from age 15 to 17, assent was obtained from the minor and informed consent was also obtained from a parent or authorized community representative. Participants were reminded about content of the consent prior to the in-depth interview to ascertain their understanding and willingness to participate in the interview. To preserve participant confidentiality, anonymous unique participant identification numbers were used throughout the study.

## Results

Forty HIV-negative AGYW ages 15–24 participated in an in-depth interview. Table 2 below describes the characteristics of the IDI participants. The majority of women interviewed were unmarried and most had at least completed primary school education. About half had a previous pregnancy and most participants reported previous condom use. Though we anticipated differences in the domains of interest between high-engagement and low-engagement participants and preferences in accessing PrEP, no differences were observed.

**Table 2:** Baseline characteristics of HIV-negative participants from in-depth interviews.

	Participants (N = 40)	
	N	%
Age		
15–19 years	21	53%
20–24 years	19	48%
Marital Status		
Single	30	75%
Married	6	15%
Separated/Divorced	4	10%
Education		
Completed Primary School	36	90%
Less than Primary School	4	10%
Ever Pregnant		
Yes	18	45%
No	22	55%
Ever Condom Use (Male and Female)		
Yes	37	93%
No	3	8%
Perceived Chance of Getting HIV in Lifetime		
No Chance	10	25%
Small Chance	11	28%
High Chance	13	33%
Don't Know	6	15%
Service uptake		
High engager	20	
Low engager	20	

### AGYW’s Knowledge of PrEP

When asked if they had ever heard of PrEP, all participants had no prior knowledge of PrEP. The interviewer ensured that participants demonstrated an understanding of PrEP before continuing with questions regarding the potential use of PrEP.

This quote below is a representative of participants’ level of understanding on PrEP following the explanation.

“*…you have said that one is supposed to take this pill every day before she is exposed [sex] and it means that the time when you will be exposed you will already have the drug in your body and the virus cannot be active in the blood* **…*”* (19 years old, unmarried, high engager)**

### AGYW’S interest in PrEP

Most AGYW expressed an interest in using PrEP. This interest was grounded in women’s perception of the severity of HIV infection and the desire for protection against HIV.

***“…*.** *if I am at risk of contracting the virus*, *then I will not contract the virus if I am on the drug*. *So I will be protected*. *You know how HIV/AIDS has no medicine*, *so it is very scary*. **(23 years old, unmarried, low engager)**

The perceived need for constant protection against HIV was also linked to the perceived likelihood and threat of rape for some women. Participants felt that PrEP would be the best way to protect themselves in situations of forced sex when they would have no control over their HIV risk exposure:

*“I can feel free to take [PrEP] because you never know what you can come across*. *Say for example*, *if thieves break in your house*, *it is we girls who are on a very high risk of being raped*. *Thieves cannot rape your brother*. *So what you have explained to me about PrEP*, *I think it is good to take it because you cannot contract HIV*.*”* **(18 years old, unmarried, high engager)**

AGYW were also interested in PrEP for HIV risk exposures in the context of their relationships. Participants discussed two primary causes of their perceived HIV risk contributing to their interest in PrEP: their own and their partners’ concurrent sexual relationships, and inconsistent condom use.

### Concurrency and risk for HIV

Most participants acknowledged that having multiple concurrent sexual partners could increase their risk of contracting HIV. This participant discusses the compounded HIV risk presented by both partner concurrency and non-condom use, demonstrating the complex risk calculus that may be involved in the perceived need for PrEP:

*“Because if I maybe*, *have a lot of partners*, *maybe someone can encourage me to say ‘I do not want us to use a condom*, *let us have unprotected sex*,*’ so that is how I can contract diseases*.*”* **(21 years old, unmarried, high engager)**

Some AGYW also worried about their partners’ risk behaviors beyond their control. AGYW worried that even though they themselves might be faithful to one partner, their partners might have other partners. This raised their interest in using PrEP to remain protected. One participant said:

“*It [PrEP] can bring about good things*. *Because say in a relationship*, *some men go behind their girlfriends and have another relationship with another girl or some may even go to the extent of having sexual relations with prostitutes*. *So you’d be protected*.*”*

(21 years old, unmarried, high engager)

This participant and others saw PrEP as an opportunity to be protected against this source of HIV risk outside of their control. PrEP was seen as beneficial not only for personal protection against HIV, but also as a potential protection benefit for a sexual network, as this participant said:

*“I like it [PrEP] because there are a lot of people that have numerous sexual partners and sleep with them without condoms except maybe one partner so it would help to protect these numerous sexual partners from getting infected*.*”***(21 years old, unmarried, low Low engager)**

Other women discussed the anxiety of not knowing the HIV-status of their sexual partner(s). Sex with these partners of unknown status would bring them fear and stress about contracting the HIV virus. They saw PrEP as something to alleviate this stress.

*“Because sometimes you can have sex with someone whose status you don’t know and that can stress you a lot thinking that you may have contracted the virus*. *However*, *if you start taking the drugs you can just know that you are safe even if you have sex with someone who is positive*.*”* **(22 years old, unmarried, low engager)**

### Inconsistent condom use and user error

Most participants discussed PrEP interest in the context of non-use or inconsistent use of condoms. Many women found PrEP appealing because it would eliminate concerns about having a condom available at every sexual encounter. As this 21-year-old, unmarried young woman said: ***“****I would be interested to use it [PrEP] because I am at risk*. *I may end up having unprotected sex with someone just because they did not have a condom*.*”* Participants connected these concerns about unplanned sexual encounters and the lack of condoms and resulting HIV exposure risk to their interest in PrEP. In addition to the issue of condoms, one participant discussed user-error in condom use as motivating factor for her interest in using PrEP:

*“PrEP is good*, *because if you might wear a condom and it gets torn without expecting it and they can give you the virus but if you had that medication even when the condom got torn you would not get the virus*.*”* **(23 years old, unmarried, low engager)**

Participants were also interested in using PrEP because they perceived condom use to be under the control of their male partners, who may dislike them. One 19-year-old, unmarried participant explained: *“I think the medicine is good because most men refuse to use condoms*.*”* The availability of PrEP would protect the AGYW under such circumstances.

One participant was interested in PrEP in the wider context of preferences for condom-less sex. She perceived that many young people would like to use PrEP to enjoy condom-less sex while still being protected from HIV acquisition.

“… *a lot of youth complain about condoms*, *that they do not feel good when having sex*. *So if they would drink the medicine accordingly they would be happy*. *Because they would know they have medicine that will protect them*.*”* **(20 years old, unmarried, high engager)**

### Reasons for disinterest and concerns about PrEP

Participants generally expressed positive attitudes toward PrEP; only five participants out of forty the AGYW interviewed, expressed no interest in PrEP. Typically, participants who perceived themselves to be at very low HIV risk were less interested in PrEP. These participants perceived it that being abstinent or having only one partner and being faithful to that partner put them at a very low risk of acquiring HIV, and were disinterested in PrEP use for that reason. As this 22-year-old, unmarried, low engager participant said: *“I’m not interested because I only have one partner*.*”*

Women also had concerns that PrEP could negatively affect their social wellbeing. Chiefly, they were concerned about partner reactions to their PrEP use, social stigma, and the need for dual protection for pregnancy prevention if using PrEP. These concerns are discussed below.

### Partner concerns

Some participants, expressed concerns about sexual partners’ potential attitudes toward their use of PrEP, especially among married young women. They worried that partners would suspect their women of having extra-marital affairs. In this regard, one unmarried woman explained:

*“There would be problems in marriage…their [married women’s] partners would question them and would suspect that they have sex with other people*. *Many men [may] think that when a woman uses that drug that means she is not faithful*, *there is something she is doing and she does not want to give her partner HIV*, *instead of understanding that the drug will help them together*.” **(20 years old, unmarried, low engager)**

As this woman expresses, a woman’s use of PrEP could be seen as harmful to the level of trust in a marital relationship, implying infidelity. In the marriage context, participants believed that a couple has to be faithful to each other, and thus women taking the initiative to take PrEP on their own might be seen to violate this norm. This young woman explained how she thought this would be perceived in a marriage:

*“… Imagine these drugs are found with a married woman*, *what picture is that going to give*? *It may mean that there is lack of faithfulness between the two*.*”* **(18 years old, married, low engager)**

### Negative community perceptions about PrEP use and side effects

Some participants were also concerned about possible community reactions to their use of PrEP. Some women felt that people might think they are HIV positive and taking ART; this woman explained her fear as follows.

*“The fear can be there because you are supposed to take this drug daily wherever you go and people may misinterpret it to think they are ARVs”* **(19 years old, unmarried, high engager)**

Apart from fears of the appearance of taking a daily pill, some women were concerned about the appearance of PrEP side effects that could be also misperceived. While most AGYW said they would consult doctors for professional help if unacceptable side effects occurred, a few said they would stop taking PrEP if the side effects could lead to negative perceptions by members of the community. One woman suggested that side effects that affect your productivity or mimic pregnancy would lead to feelings of shame, stigma, and social rejection.

*“… I would not be at peace with them [PrEP pills]*, *because when you take something and you start having other problems that means you won’t be able to work*. *What if you go to the market and fall at the market people will be wondering if you are pregnant or what…”* (**20 years old, unmarried, low engager)**

In addition to being concerned about community reactions, some women also expressed their own stigmatizing attitudes toward PrEP use. Participants felt that the confidence in being protected against HIV provided by PrEP would promote behaviors that they perceived as “promiscuous” in users:

“*What I am trying to say is that PrEP will give room to promiscuity; people on PrEP will sleep around a lot just because they know that they have this [protection]*,*”* **(18 years old, married, low engager).**

### Need for dual protection

Women were also concerned that PrEP users who no longer felt the need to use condoms would not be sufficiently protected against pregnancy and other sexually transmitted infections (STIs). They expressed the disadvantage of PrEP as compared to condoms for those women hoping to avoid pregnancy, since both PrEP and contraception would have to be used in combination to prevent pregnancy and HIV. One 22-year-old, unmarried, low engager commented: *“Condoms are better because with PrEP*, *you only protect yourself against HIV but you can become pregnant while with condoms you are protected against both pregnancy and infection*.*”* The failure of PrEP to protect young women from pregnancy and other STIs, and the burden to protect themselves against unplanned pregnancy and other STIs made women less interested in using PrEP.

### Preferences for PrEP delivery

On balance, AGYW were generally interested in PrEP, despite the concerns expressed above. Women, however explained that that their interest in initiating PrEP would also depend upon the ease of accessing PrEP, packaging attributes, and delivery context. Participants commonly held the opinion that the process of obtaining pills should be confidential and the appearance of PrEP packaging should be discrete to avoid negative perceptions.

### Packaging preferences

Participants shared a variety of ideas for PrEP packaging that would boost their interest. They favored PrEP to be packaged in cartons, packets, or bottles that would give the appearance of medications for common ailments. Particularly, AGYW emphasized that PrEP packaging should not resemble antiretroviral drugs to avoid the perception of receiving treatment for HIV and being subjected to the stigmatization associated with this. This 21-year-old, unmarried, high engager participant explained: “*[They should be packaged] in papers [small plastic zip lock bags]*, *because if they are put in bottles people will say ‘iii that person*,*’ they will think you are taking ARVs*, *when you aren’t*. *But when they are in papers people think it’s just these other medicines*. If PrEP drugs were the same antiretroviral drugs that are used for HIV treatment, women were concerned that being seen with ARVs would lead others to believe that they are HIV-positive.

In addition to the desire to avoid the appearance of being treated for HIV, women further preferred that the packaging be discrete enough but “feminine,” resembling drugs that women routinely use for contraception to keep their use of PrEP confidential. As one participant put it: “*Maybe they should be packaged like contraceptive pills and not like ARVs so that not everyone should know [that I am using PrEP]*,*”* (19 years old, unmarried, high engager).

### Dispensing location preferences

Most participants suggested having PrEP available in locations frequented by youth such as schools would be appealing. Reasons for this included ease of access and comfort in the absence of adult patients and family members. One participant explained:

*“Because most of the youth or let’s say three quarters of the youth we meet at school* … *As such*, *it can be good if these drugs are received in schools*. *Because if we take into consideration the church there is a threat because the family are also found there so if they can say all youth should remain behind because there are some drugs to be distributed*, *with the parents [present] it cannot be a good thing but rather at school where we are mostly youth*.*”* **(18 years old, unmarried, low engager)**

Other women suggested these services should be offered in youth-friendly spaces like those established in the Girl Power study. These clinics were set aside from the main facility and from adult patients, staffed by providers trained in youth-friendly service delivery, and had young clinic navigators who assisted AGYW access services. This woman explains her preference: “*A place for the youths to be comfortable getting these drugs*, *like this one [Girl Power clinic] it’s just the two of us [the provider and I] in here without any problem*,*”* (22 years old, married, high engager).

Other participants suggested similar youth-friendly models, sharing the opinion that it would be beneficial to receive integrated sexual and reproductive health services in tandem with PrEP services:

*“… We could set aside a room where many youths would go access this drug*. *It would be appropriate to offer the drug where the other services like social harms or family planning services are offered*. *Therefore*, *if there would be a special room where someone would be tested for HIV and given the drug after*, *then people would be assisted easily*.*”* **(20 years old, unmarried, low engager)**

## Discussion

While there is increasing evidence that PrEP is safe and efficacious in preventing HIV in diverse populations [13], little is known about the acceptability of and interest in PrEP among adolescent girls and young women (AGYW) in sub-Saharan Africa. Women in this study were generally interested in PrEP, and this interest was grounded in a belief that much of their HIV risk exposure was due to factors that were out of their control, including partners having concurrent relationships, challenges with condom use, and rape. These findings are consistent with other studies describing AGYW’s interest in emerging biomedical HIV prevention technologies [14]. Women found the consistent protection of PrEP to be appealing for these reasons, but those women who perceived their HIV risk to be low were less interested in it. Women were further concerned that the appearance of PrEP packaging and side effects could lead to negative community perceptions including perceived promiscuity. For these reasons, women recommended that PrEP be packaged discretely and not carry the appearance of ART. Finally, women suggested that PrEP should be delivered in youth-friendly settings providing access to PrEP care, education, and counseling tailored to the youth.

None of the women participating in this study had ever heard of PrEP, but the majority were interested in using PrEP after learning about its efficacy and safety. High acceptability of PrEP has been found in similar populations of AGYW [15], and in other populations of adult women in sub-Saharan Africa. Pregnant and postpartum young women in a study in South Africa were much more interested in PrEP upon learning about its efficacy, and studies of other populations of potential PrEP users suggest that PrEP’s acceptability is greatly improved with education about its efficacy in preventing the acquisition of HIV [16,17]. This suggests that promoting women’s knowledge of PrEP’s effectiveness will be important in promoting interest in this prevention technology, and may be critical to help potential users make informed choices about using PrEP.

The high level of hypothetical PrEP acceptability among AGYW in our study suggests that there is potential interest in its use. Many AGYW in our study found PrEP appealing because its use is less sex-act dependent and more personally controlled than condoms. Using PrEP as an alternative approach to condoms has potential implications for other sexually transmitted infections (STIs) and unintended pregnancy, something noted by our participants. Once implemented, the effect of PrEP availability on these outcomes should be monitored and evaluated. Despite the importance of monitoring this potential unintended consequence of PrEP use, previous evidence suggests that PrEP use does not necessarily lead to risk compensation and cessation of condom use, but rather enhances informed choices [18].

HIV risk awareness may enhance AGYW’s interest in and uptake of PrEP. In this study, women’s interest in PrEP was highly dependent upon their perceived level of HIV risk. Although some AGYW did not perceive themselves to be at risk of HIV infection, many of the same women did express a concern about the possibility of their partners having other sexual relationships. These findings echo previous studies, which suggest that AGYW may not accurately perceive their HIV risk [19]. In the FEM-PrEP trial, half the sero-conversions happened in individuals who perceived themselves to be at low risk for HIV[20,21]. Together these findings suggest that raising awareness of PrEP may need to be coupled with counseling on potential sources of HIV risk. This finding highlights the need to offer AGYW comprehensive sexual and reproductive health services to help them better assess their HIV risk and make informed decisions about the use of PrEP and other HIV prevention products[22].

Our findings also corroborate previous evidence which suggests that offering PrEP in a youth-friendly environment may promote AGYW’s interest in initiating PrEP [23,24]. Most of our interviews were conducted among participants who were able to receive other sexual and reproductive health services in a youth-friendly environment in the context of Girl Power, and described features of this environment as appealing for PrEP distribution as well. Although participants had mixed suggestions for the specific location in which to offer PrEP (schools, hospitals, clinics), they emphasized having a separate youth-friendly space for PrEP and other sexual and reproductive health care service away from adults.

In addition to offering PrEP in a youth-friendly environment, participants recommended that PrEP packaging be discrete. Most participants wanted PrEP packaging to appear distinct from that of typical ART packaging to maintain privacy and avoid the appearance of being treated for HIV. While discrete packaging may be a short-term approach to mitigating HIV and PrEP-related stigma, addressing these issues more directly could also be important to facilitate AGYW’s utilization of PrEP. Our findings are consistent with those of a study of AGYW in Kenya [25], which similarly found that discrete packaging of PrEP could serve to mitigate concerns about stigmatization and consequently motivate PrEP use. The issue of packaging to ensure integrity, safety and effectiveness of medications is important and should be taken into account when planning PrEP interventions [22]. Related to concerns about stigma, helping women understand and manage PrEP-related side effects could also increase the acceptability of PrEP among AGYW.

### Limitations

The results of our study should be interpreted with limitations in mind. Participants lacked prior knowledge and experience with PrEP use, thus their responses to questions about their potential interest in PrEP were based on limited information and consideration. Some concerns raised were hypothetical such as certain side effects, and might not bear out in the real world. Because participants were enrolled in a study in which youth friendly services for HIV prevention were offered as part of the study intervention, their positive impressions of a youth-friendly context may reflect social desirability. AGYW were recruited for participation in the larger trial on a convenience basis through community outreach, participant referral, and self-referral from urban and peri-urban settings in Lilongwe; as such, the study sample may not be representative of the target population of sexually active AGYW in the clinic catchment areas, or of AGYW living in rural areas or other regions of Malawi.

Despite these limitations, the findings of this study suggest that PrEP may be an acceptable method for HIV prevention among sexually active AGYW in Malawi. As PrEP is introduced in Malawi, it is important to ensure adequate sensitization for the women themselves, their partners, and communities. Furthermore, its successful introduction may hinge on how PrEP is offered—the way it is packaged and whether it is delivered in an environment that is sensitive to AGYW concerns. These findings can meaningfully inform the implementation of this new program for this high-risk population.
